# Using Centromere Mediated Genome Elimination to Elucidate the Functional Redundancy of Candidate Telomere Binding Proteins in *Arabidopsis thaliana*

**DOI:** 10.3389/fgene.2015.00349

**Published:** 2016-01-05

**Authors:** Nick Fulcher, Karel Riha

**Affiliations:** ^1^Gregor Mendel Institute, Austrian Academy of Sciences, ViennaAustria; ^2^Central European Institute of Technology, Masaryk University, BrnoCzech Republic

**Keywords:** telomeres, centromere, haploid, telobox, protein family

## Abstract

Proteins that bind to telomeric DNA form the key structural and functional constituents of telomeres. While telomere binding proteins have been described in the majority of organisms, their identity in plants remains unknown. Several protein families containing a telomere binding motif known as the telobox have been previously described in *Arabidopsis thaliana*. Nonetheless, functional evidence for their involvement at telomeres has not been obtained, likely due to functional redundancy. Here we performed genetic analysis on the TRF-like family consisting of six proteins (TRB1, TRP1, TRFL1, TRFL2, TRFL4, and TRF9) which have previously shown to bind telomeric DNA *in vitro*. We used haploid genetics to create multiple knock-out plants deficient for all six proteins of this gene family. These plants did not exhibit changes in telomere length, or phenotypes associated with telomere dysfunction. This data demonstrates that this telobox protein family is not involved in telomere maintenance in *Arabidopsis*. Phylogenetic analysis in major plant lineages revealed early diversification of telobox proteins families indicating that telomere function may be associated with other telobox proteins.

## Introduction

Telomeres represent the nucleoprotein complexes that cap natural chromosome ends and function in the suppression of DNA damage signaling and control of cellular senescence. The classical telomere structure comprises tandem arrays of TTAGG-like sequences which contain G-rich 3′ overhangs at their termini. TRF1 and TRF2 represent the core duplex binding proteins of the mammalian telomere capping complex known as shelterin (de Lange, 2005); TRF1 is thought to be a regulator of telomere length (van Steensel and de Lange, 1997) and TRF2 has been shown to play a central role in protecting chromosome ends from end to end fusions and recombination (van Steensel et al., 1998; Wang et al., 2004). In contrast to the situation in a number of eukaryotic organisms which have extensively characterized chromosome-end capping protein complexes, the plant telomere binding components remain elusive (Watson and Riha, 2010). A hallmark of telomere binding proteins includes the presence of a single Myb domain containing the telobox, a motif that provides specificity to the telomeric sequence (Bilaud et al., 1996). Telobox containing proteins (TRF-like, TRFL) are present in genomes of all major groups of eukaryotes and they have been considered the prime suspects for *bona fide* telomere binding proteins in plants. Indeed, functional analysis of TRFL proteins in rice and tobacco has indicated their involvement in telomere length homeostasis (Yang et al., 2004; Hong et al., 2007).

TRFL proteins have been extensively studied in *Arabidopsis*. The *Arabidopsis thaliana* genome encodes at least 15 proteins containing a single Myb domain with the telobox that are divided into three families (Zellinger and Riha, 2007). The Smh/TRB family consists of three proteins harboring a histone H1-like motif involved in multimerization, and the Myb domain at the N-terminus (Marian et al., 2003; Kuchar and Fajkus, 2004; Mozgova et al., 2008). The second family includes six proteins (TRFL3, 5, 6, 7, 8, 10; TRFL Group II) that are unable to bind telomeric DNA *in vitro*, and are also unable to form homo- and heterodimers, despite possessing the C-terminal Myb-telobox domain (Karamysheva et al., 2004). The third family also consists of six proteins with the C-terminal Myb domain (TBP1, TRP1, TRFL1, TRFL2, TRFL4 and TRFL9; TRFL Group I), but these proteins homo- and heterodimerize and can efficiently bind to telomeric DNA *in vitro* (Karamysheva et al., 2004). A key feature of this family is a ∼30 amino acid extension of the Myb-telobox domain that is likely responsible for specific binding to plant telomeric DNA. Structural studies of related tobacco and rice TRFL proteins determined that their binding to telomeric DNA occurs in a similar fashion as for human TRF1 (Ko et al., 2008, 2009). Thus, members of the TRFL Group I family have long been considered to act as putative telomere binding proteins in *Arabidopsis*. Nevertheless, plants containing single knockouts within members of this gene family have not shown drastic telomeric phenotypes (Karamysheva et al., 2004). The lack of severe telomere related phenotypes similar to mammalian TRF2 knock-outs suggested a functional redundancy among these proteins in *Arabidopsis*.

Reverse genetics based approaches have been used over many studies in *Arabidopsis* to target functional redundancy amongst gene families. Construction of lines with multiple T-DNA insertions in desired genes can, however, be time consuming requiring extensive genotyping of large populations of recombinant plants. Methods to improve the production of such mutant lines would be greatly beneficial to elucidate functional redundancy within gene families. Centromere mediated genome elimination has proven to be a powerful tool in *Arabidopsis* genetics allowing generation of haploid plants, rapid production of recombinant inbred lines, and reverse breeding approaches (Ravi and Chan, 2010; Seymour et al., 2012; Wijnker et al., 2012; Ravi et al., 2014). Crossing fertile male plants to the female *cenh3*/GFP-*tailswap* haploid inducer allows for the segregation of haploid plants containing genomes from the male parent. This technology also has the potential to easily generate multiple homozygous mutant combinations when crossing plants segregating for numerous T-DNA insertions to the haploid inducer (Ravi et al., 2014). In this case, haploid plants with interesting combinations can be analyzed directly for phenotypic defects, or diploids can also be recovered in the next generation due to spontaneous diploidization. This process would greatly reduce the genotyping workload that is normally associated with the generation of quadruple or sextuple mutants by selfing alone.

In this study we tackled the functional redundancy thought to occur in *Arabidopsis* TRFL Group I family by using production of haploid plants via centromere mediated genome elimination. We have demonstrated that this method substantially facilitates generation of multiple quadruple, quintuple and sextuple mutants. Surprisingly, results show that multiple mutants do not display drastic telomeric length defects as shown for the mutants in other genes known to act at telomeres. This demonstrates that, at least in *Arabidopsis*, the TRFL protein family harboring the Myb extension does not contribute to telomere protection and/or maintenance. Furthermore, this study shows another use for centromere mediated genome elimination in the production of lines containing multiple mutations.

## Materials and Methods

### Plant Lines

All T-DNA insertions used are shown in Supplementary Table S1 and Supplementary Figure S1. The *tbp1-1* mutant was obtained from the Institut National de la Recherche Agronomique Versailles (INRAV) collection and other alleles were from the European *Arabidopsis* stock centre (NASC). Plants were grown at 22°C in 16 h light/8 h dark cycles.

### Centromere Mediated Genome Elimination

The *cenh3*/GFP-*tailswap* haploid inducer line was described previously by (Ravi and Chan, 2010). Homozygous *cenh3* mutant plants were confirmed by PCR genotyping using derived Cleaved Amplified Polymorphic Sequence (dCAPS) oligos (5′-GGTGCGATTTCTCCAGCA GTAAAAATC-3′ and 5′-CTGAGAAGATGAAGCACCGGCGATAT-3′). Resulting PCR products were digested with EcoRV, cleaved wild type (WT) alleles produced 191 and 24 bp fragments.

Haploid inducer *cenh3*/GFP-*tailswap* lines are mostly male sterile, but can be crossed as female. Heterozygous quadruple or sextuple mutants were crossed to *cenh3*/GFP-*tailswap* lines to produce haploid offspring that were homozygous for a combination of insertions derived from the male parent. Only plants that displayed the haploid phenotype as described by Ravi and Chan (2010) were selected for further analysis. These haploids were then subject to PCR genotyping using oligos shown in Supplementary Table S1. Diploid seeds can then be recovered from haploid plants due to spontaneous diploidization which allowed analysis of subsequent generations.

### DNA Extraction and Telomere Analysis

One to two leaves were homogenized in 500 μl Extraction buffer (0.2 M Tris pH9, 0.3 M LiCl, 25 mM EDTA, and 1% SDS) tubes were centrifuged for 10 min at 4000 rpm (rcf 1756 *g*) and 350 μl was transferred to 350 μl isopropanol. Tubes were inverted to mix and centrifuged for 20 min at 4000 rpm. Supernatant was poured away and the pellet was washed with 70% Ethanol. The remaining pellet was air dried and resuspended in 100 μl dH_2_O. Telomere length was determined by terminal restriction fragment analysis, and statistical analysis of telomeric smears was performed using the TeloTool software (Gohring et al., 2014; Fulcher et al., 2015). Integrity of blunt ended telomeres was determined as previously described (Kazda et al., 2012).

### Phylogenetic Analysis

Sequences of telobox containing proteins were obtained from indicated plant genomes by using http://www.phytozome.net, protein BLAST searches with the *A. thaliana* TRFL6 protein sequence as a query. Proteins were aligned by the ClustalW method and phylogenetic trees were constructed by Neighbor Joining method using CLC Main Workbench software (Qiagen).

## Results

### Knockouts of TBP1 and TRFL9 Showed No Changes in Telomere Length and Blunt End Distribution

**FIGURE 1:**
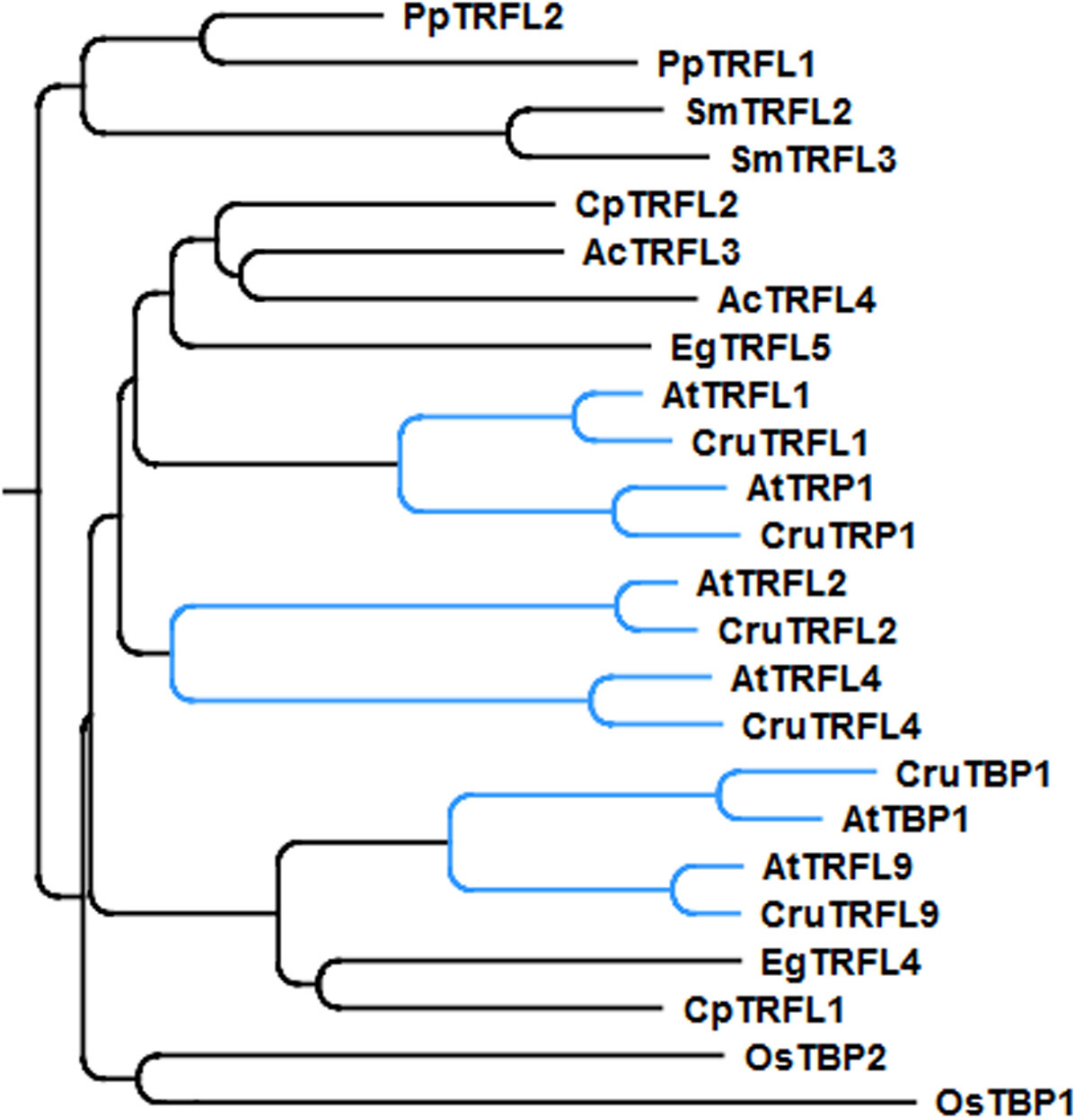
**Phylogenetic tree of the Group I TRFLs (Ac, *Aquilegia coerulea*; At, *Arabidopsis thaliana*; Cp, *Carica papaya*; Cr, *Capsella rubella*; Eg, *Eucalyptus grandis*; Os, *Oryza sativa*; Pp, *Physcomitrella patens*; Sm, *Selaginella moellendorffii*).** Clade *Brasicaceae* is indicated in blue.

**FIGURE 2:**
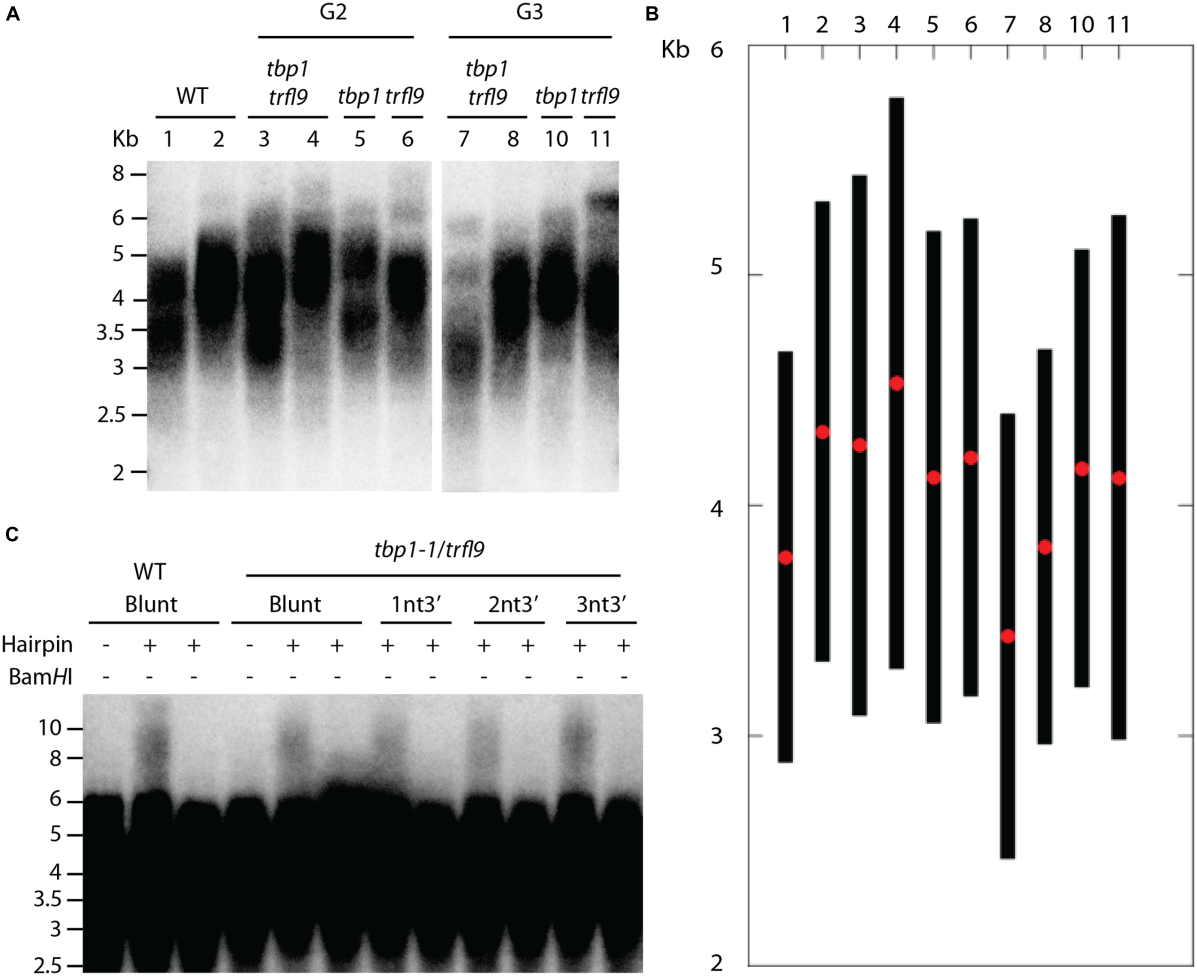
**Telomere analysis of single and double tbp1 and *trfl9* mutants. (A)** TRF blot showing telomere lengths of second and third generation *tbp1* and *trfl9* single and double mutants. Heterozygous tbp1 and trfl9 plants were crossed and wild type (WT), single, and double mutants were segregated. WT and double mutant samples show two biological replicates. Corresponding lanes from both generations show plants derived from the same lineage. Data from this blot was extracted using the TeloTool software, a representative graph is shown in **(B)**. Red dots represent the extracted mean of the smear and black bars represent calculated range values. **(C)** Double mutants for *tbp1 trfl9* were also subject to blunt end telomere analysis which showed no change in the distribution of blunt ended telomeres.

Phylogenetic analysis indicated that *A. thaliana* Group I TRFL proteins result from relatively recent duplication events in *Brasicaceae* (**Figure 1**). Therefore, some paralogs may still retain overlapping functions. To begin elucidating the role of TRFL proteins at telomeres, we first examined the published allele of *tbp1-1* which has been reported to show telomere elongation (Hwang and Cho, 2007). Within the TRFL family, TBP1 contains a closely related family member, TRFL9, which displays a high level of sequence conservation (**Figure 1**). We reasoned that double knockouts could exacerbate *tbp1-1* telomere phenotypes. Heterozygous plants containing the published *tbp1-1* allele (FLAG_072C05) were crossed to plants heterozygous for the *trfl9* (GK-036D11) mutation. Double heterozygous F1 plants were then selfed and First generation WT, double, and single mutants were segregated. DNA from five pooled plants was extracted from second and third generation of double mutants of the same lineage and subject to TRF analysis (**Figure 2**). To extract data from TRF blots, we used the recently published software TeloTool to measure telomere length and create graphs to better illustrate mean and range of telomeric smears (Gohring et al., 2014). No difference in telomere length was observed in second and third generation *tbp1-1* mutants compared to WT plants segregated from the same cross (**Figures 2A,B**). Double *tbp1 trfl9* mutants also did not appear to shown any great change in telomere length over three generations. Previous studies have shown that telomere lengthening occurs gradually in *tbp1-1* mutants over four generations (Hwang and Cho, 2007). Mutants for telomerase were also shown to show a loss of telomeric DNA of approximately 500bp per generation along with displaying a discrete banding pattern (Riha et al., 2001). However, it would be expected that knocking out core telomere associated proteins would lead to an immediate and severe effect. This has been shown in many studies where severe telomere defects were observed in *Ku70*, *stn1*, *ctc1*, and DNA polymerase α mutants, these are observed within one generation (Riha et al., 2002; Song et al., 2008; Surovtseva et al., 2009; Derboven et al., 2014).

We further examined telomere-end structure as depletion of telomere binding proteins may impair chromosome end protection and integrity of blunt-ended telomeres that are present in plants (Kazda et al., 2012). The current model for chromosome end protection in *Arabidopsis* suggests that telomeres at the leading end are protected from nucleotytic processing by the Ku heterodimer immediately after DNA replication. Because of this, lagging end telomeres in plants are thought to generate classical T-loop structures, whereas leading end telomeres remain blunt-ended and protected by Ku. A hairpin ligation assay was previously developed by Kazda et al. (2012) to detect the presence of blunt ends at *Arabidopsis* telomeres. Briefly, hairpin sequences containing a *Bam*HI site are ligated to blunt-ended telomeres and DNA is digested with *Alu*I to liberate telomeres. Hairpin structures are then subject to alkaline electrophoresis which produces a shift in the higher molecular weight TRF signal. Digestion with *Bam*HI shows that these events are the result of ligation of the hairpin to natural telomeric ends.

Because of the essential role of telomere binding proteins in telomere protection, we reasoned that their inactivation would lead to resection of blunt ended telomeres. However, no observable difference was seen in the presence of blunt ends in *tbp1 trfl9* double mutants using blunt end and short-overhang containing hairpins (**Figure 2C**). These data argue that absence of TBP1 and TRFL9 does not have any discernible effect on telomere structure.

### Multiple Combinations of Quadruple, Quintuple, and Sextuple Mutants Showed No Large Effect on Telomere Length

Because of the sequence similarities between the TRFL proteins, it is possible that other TRFL homologs compensate the functions of TBP1 and TRFL9 in their absence. Therefore, we decided to construct *Arabidopsis* plants with multiple mutant combinations of the genes in the group I TRFL family. Because generation of sextuple mutants would require extensive screening of a large number of plants in segregating populations, we decided to take advantage of centromere induced genome elimination to produce haploid F2 plants (Ravi and Chan, 2010). Frequency of any quadruple mutant combination among such haploids is 1/16 as opposed to 1/256 in a diploid F2 population.

**FIGURE 3:**
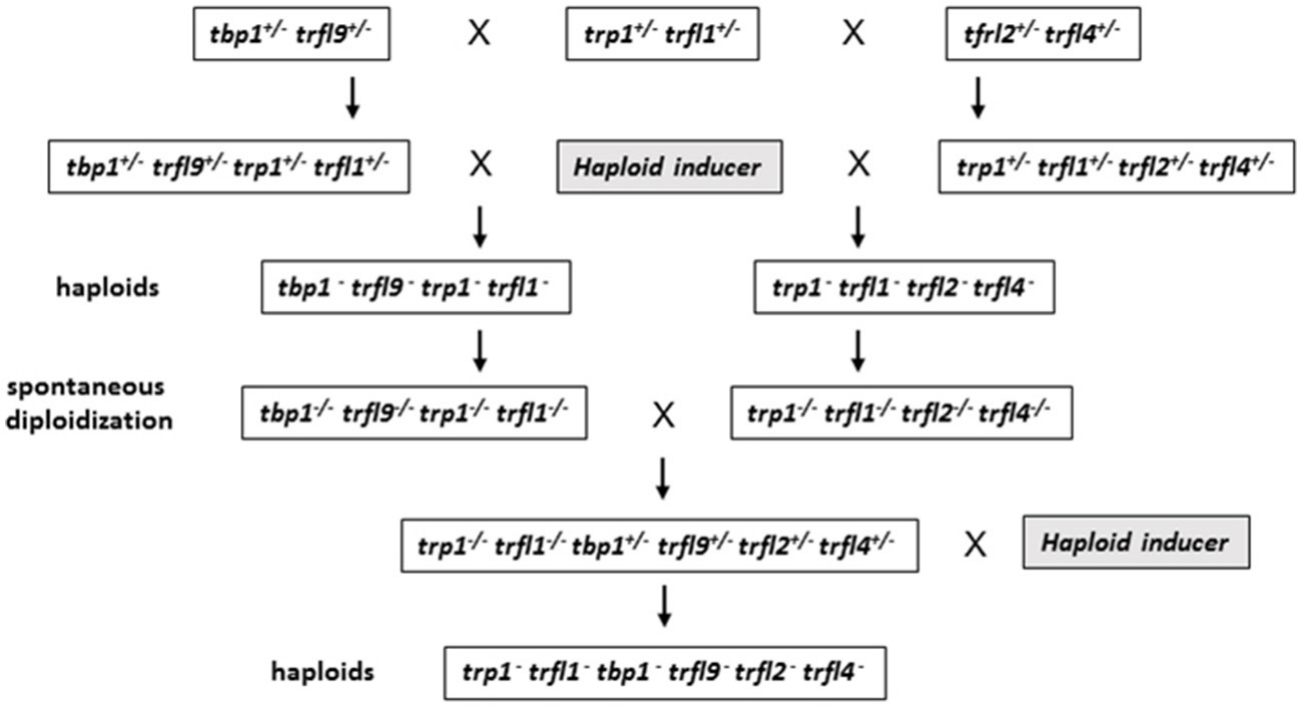
**Diagram illustrating crossing scheme for generating multiple TRFL mutants by using centromere mediated genome elimination.** See text for details.

**FIGURE 4:**
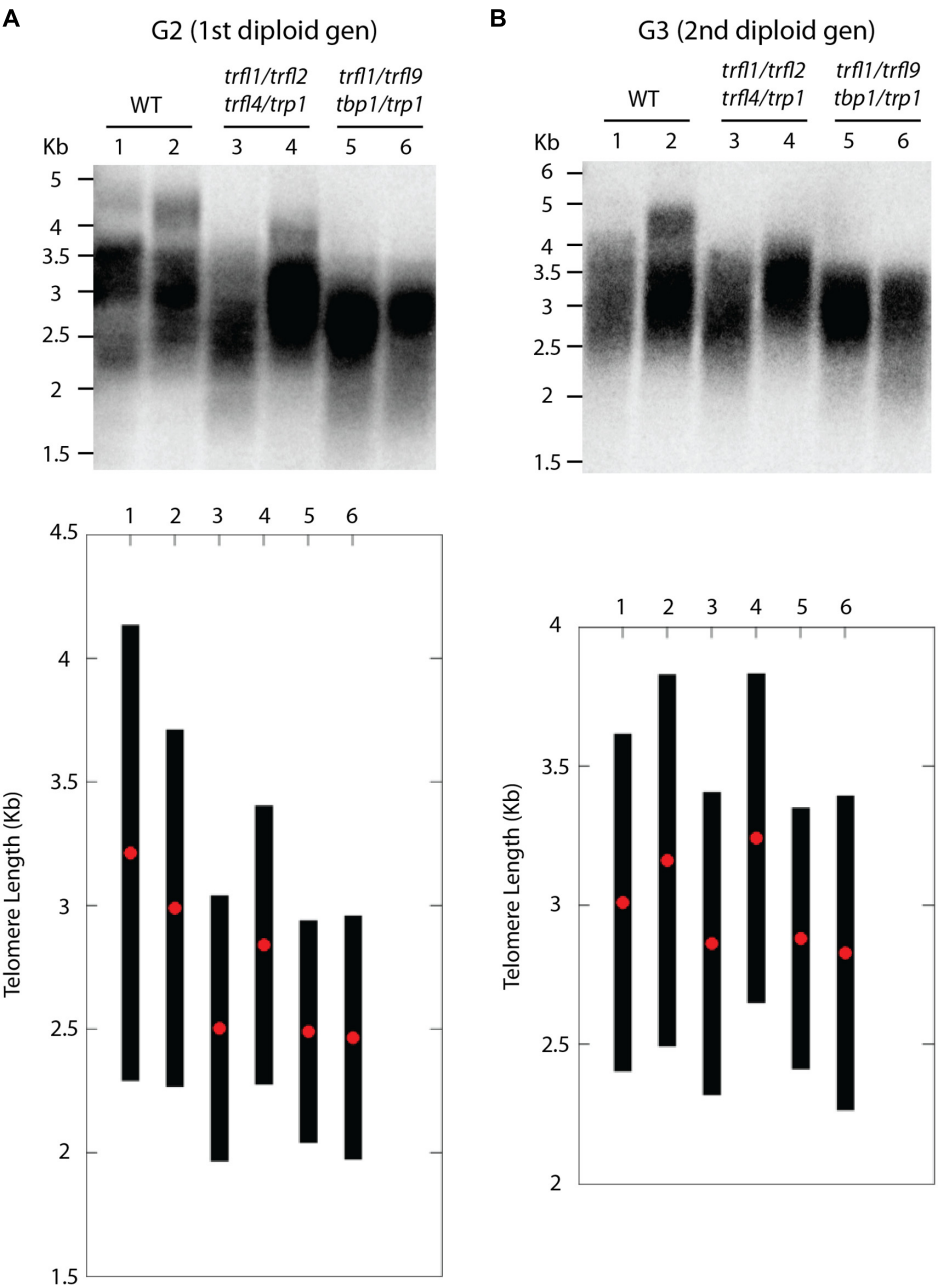
**Telomere length analysis of quadruple *trfl1 trfl2 trfl4 trp1* and *trfl1 trfl9 tbp1 trp1* mutants.** TRF blots show telomere lengths from second generation **(A)** and third generation **(B)** plants along with WT controls. Blots from the Second generation show TRF profiles of two individual doubled haploid plants. DNA was pooled from 5 plants in the next generation and subject to TRF analysis in the third generation. Corresponding lanes of both blots represent plants of the same lineage. Graphs plotted from TeloTool analysis are shown below respective blots. Red dots represent the extracted mean and black bars show calculated range values.

Centromere induced genome elimination involves generation of haploids by crossing diploid plants as male to the *cenh3*/GFP-*tailswap* haploid inducer. Single T-DNA insertion mutants were selected for each of the six candidate proteins. In addition to *trfl9* and *tbp1* alleles which were already mentioned, *trp1* (SALK_125033), *trfl1* (SALK_052864), *trfl2* (SAIL_73_G01), and *trfl4* (SAIL_73_F07) mutants were also obtained. In order to combine all alleles into the same plant, we first created three combinations of double heterozygous mutants (*trp1^++/-^ trfl1^+/-^*, *trfl2^+/-^ trfl4^+/-^*, and *tbp1^+/-^ trfl9^+/-^*). Next, we generated two combinations of quadruple mutants, and finally quintuple and sextuple mutants as illustrated in the crossing scheme (**Figure 3**). Heterozygous quadruple mutants were then crossed as male to *cenh3*/GFP-*tailswap* plants; homozygous quadruple haploids were obtained along with the WT combination. Diploid seeds were obtained from mutant and WT haploids by spontaneous diploidization (**Figure 3**). First, two single doubled haploid plants were tested by TRF analysis for WT, *tbp1^-/-^ trfl9^-/-^ trp1^-/-^ trfl1^-/-^* and *trp1^-/-^ trfl1^-/-^ trfl2^-/-^ trfl4^-/-^* combinations (Second generation without functional protein, **Figure 4A**). Seeds were collected from these plants and pooled DNA from 5 plants was used for TRF analysis in the following generation (Third generation, **Figure 4B**). Terminal restriction fragment analysis of resulting *tbp1^-/-^ trfl9^-/-^ trp1^-/-^ trfl1^-/-^* and *trp1^-/-^ trfl1^-/-^ trfl2^-/-^ trfl4^-/-^* lines showed no effect on telomere length regulation (**Figure 4**).

**FIGURE 5:**
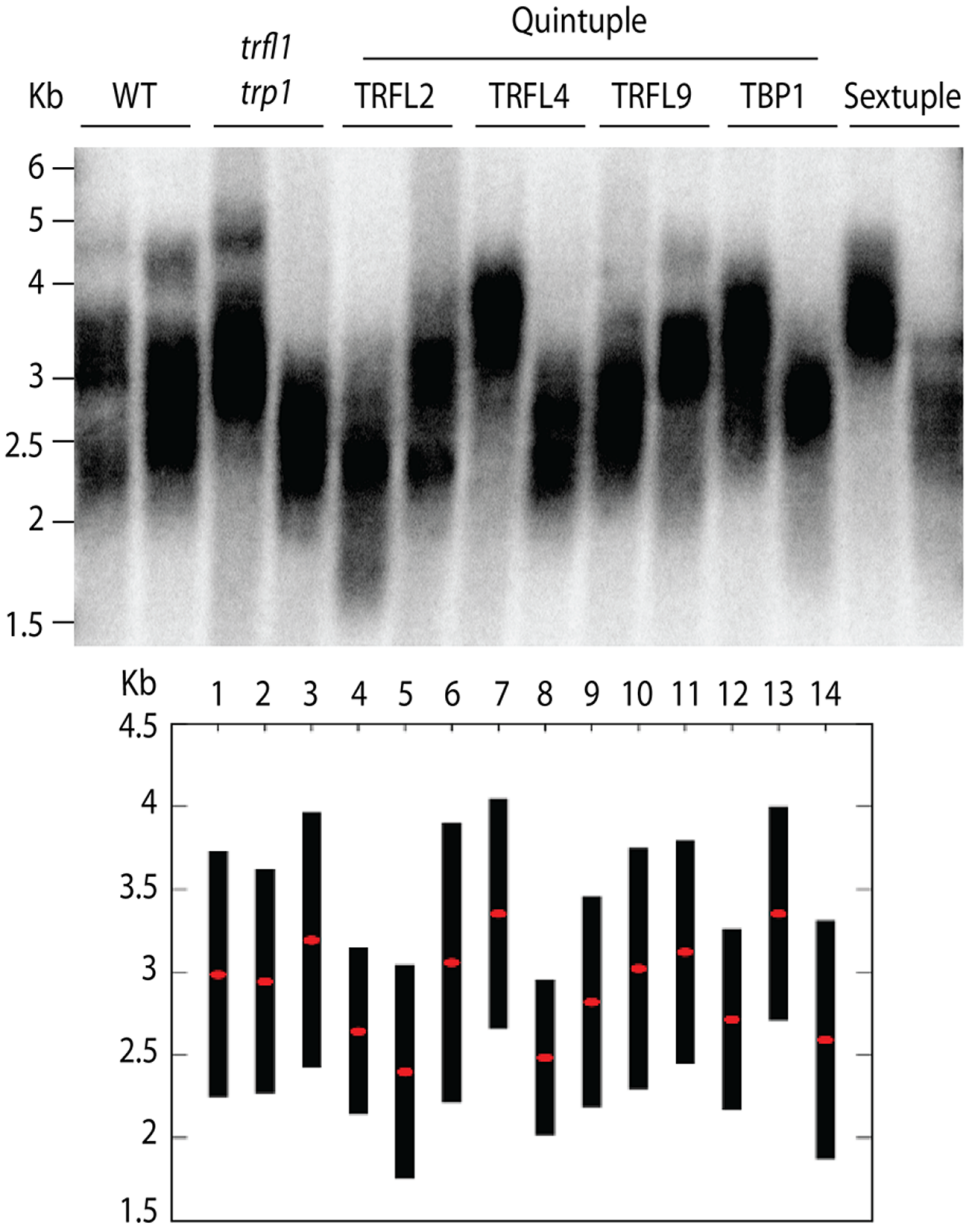
**Telomere length analysis of quintuple and sextuple mutants.** TRF blot **(upper panel)** shows telomeric smears from WT, double *trfl1 trp1*, quintuple (noted gene represents WT locus) and complete sextuple knockouts. DNA was extracted from single segregated haploid plants. TeloTool analysis is shown **(lower panel)** where red dots show extrapolated mean and black bars shown range values.

Next, we created lines with disruptions in the entire gene family. For this, both quadruple homozygous mutant lines were crossed generating F1 plants that were homozygous for *trp1 trfl1* mutations, but segregating for the other four alleles (**Figure 3**). The haploid induction process was repeated by crossing these plants to the *cenh3*/GFP-*tailswap* plants and segregating quintuple and sextuple haploid plants. Individual quintuple and sextuple haploid plants were fully viable and exhibited neither retarded growth in comparison to haploid plants that segregated as WT, nor defects typical for plants with dysfunctional telomeres (Riha et al., 2001; Surovtseva et al., 2009; Derboven et al., 2014). TRF analysis did not reveal drastic changes in telomere length in these mutants (**Figure 5**), although observed variation seen among individual samples suggests that sextuple mutants could display a higher level of telomere length variation compared to WT. The telomere lengths observed here, however, all lie within the natural telomere length limits seen in Col-0 lines and natural variation amongst diverse *Arabidopsis* accessions was also shown to vary between approximately 1 and 9 kb (Fulcher et al., 2015). Normal growth and lack of a clear telomere length deviation in sextuple mutants demonstrates that the Group II TRFL protein family does not play a major role in telomere maintenance in *A. thaliana*.

### Phylogeny of Telobox Containing Proteins in the Plant Kingdom

**FIGURE 6:**
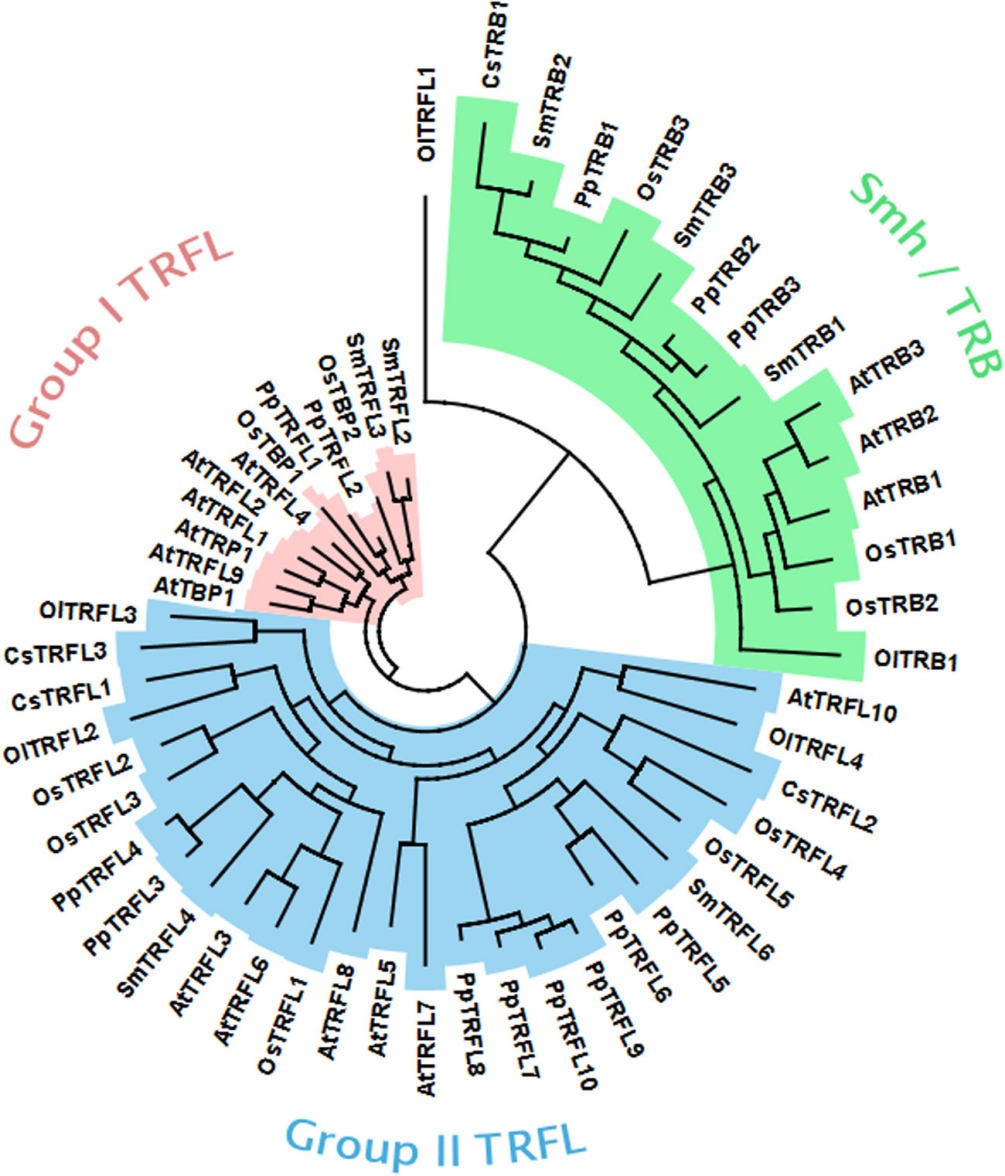
**Phylogenetic tree of plant telobox protiens. (At, *Arabidopsis thaliana*; Cc, *Coccomyxa subellipsoidea*; Ol, *Ostreococcus lucimarinus*; Os, *Oryza sativa*; Pp, *Physcomitrella patens*; Sm, *Selaginella moellendorffii*)**.

Our genetic analysis excluded the possibility that the Group I TRFL protein family harbors functional counterparts of human TRF1/2. Thus, the candidate protein(s) may be encoded by one of the other two telobox families. It is expected that that the *bona fide* telomere binding protein will be highly conserved in plants. To look at evolution of telobox protein families, we performed systematic phylogenetic analysis of all telobox containing proteins in sequenced genomes representing different phylogenetic groups within plant kingdom. In this analysis we included *A. thaliana* and *Oryza sativa* as representatives of dicot and monocot angiosperm plants, respectively, *Selaginella moellendorffii* representing the oldest branch in the clade of vascular plants, moss *Physcomitrella patens* and two unicellular green algae, *Coccomyxa subellipsoidea* and *Ostreococcus lucimarinus*. Phylogeny based on whole protein alignments revealed presence of the all three telobox protein families already in the moss *P. patens* and separation of TRFL and Smh/TRB is apparent already in unicellular algae (**Figure 6**). This demonstrates ancient origin of the three telobox protein families and their diversification early in evolution of the plant lineage. Hence, telomere function can be associated with either of the remaining two telobox families.

## Discussion

Homologs of TRF1 and TRF2, the double stranded telomere binding proteins central to the shelterin complex, have not been clearly characterized so far in *Arabidopsis*. These proteins form the core part of shelterin and are essential for telomere maintenance and function. Cells expressing dominant negative alleles and conditional knockouts of TRF2 exhibit telomere fusions and telomere length defects demonstrating an essential role of TRF2 in telomere protection (van Steensel et al., 1998; Celli and de Lange, 2005). Functional studies of TRF1 indicate a role of the protein in telomere replication and length regulation (van Steensel and de Lange, 1997; Sfeir et al., 2009) TRFL proteins described in *Arabidopsis* highlighted a group of potential candidates containing C-terminal telobox motif and plant specific extension domain (Karamysheva et al., 2004). These proteins also bind to telomeric DNA *in vitro* and the telobox domain is important for this interaction. In addition, studies have shown that disruption of similar proteins in rice, tobacco and tomato leads to telomeric and developmental phenotypes. Transformation of tobacco BY2 cells with 35S:*LeTBP1* from tomato was reported to result in telomere shortening from 15–55 kbps to 15–35 kbps (Moriguchi et al., 2006). In a later study, knockdowns of LeTBP1 in tomato showed defects in fruit development and genomic instability, no changes in telomere length were observed in these plants (Moriguchi et al., 2011). It could be, however, that in these studies, the TRF assay is not sensitive enough to detect small changes that occur in the already long telomeres of tobacco and tomato. Characterization of RICE TELOMERE BINDING PROTEIN1 (RTBP1) showed telomere elongation in first generation RTBP1 knockouts along with anaphase bridges, growth retardation, and floral defects in later generations (Hong et al., 2007). A similar result was reported in *Arabidopsis* showing knockouts of AtTBP1 undergoing telomere elongation over four generations (Hwang and Cho, 2007). However, the presence of *tbp1-1* in the Ws background complicates telomere length analysis as this accession has previously shown to display a bimodal telomere length distribution in WT plants (Shakirov and Shippen, 2004). Because of these previously reported phenotypes of these candidate telomere binding proteins in *Arabidopsis* and other plant species, *in vitro* telomeric duplex binding activity, and the high level of sequence conservation, it was expected that the Group I TRFL family comprises the canonical duplex telomere binding proteins.

However, in this study we show that knockouts of all six members of the family in *Arabidopsis* do not exhibit any obvious changes in telomere length or functionality. Thus, it can be concluded that, at least in *Arabidopsis*, Group I TRFL family does not play a major role in telomere biology. The previously reported *in vitro* telomere binding of this group suggests there is association with telomeric DNA, although an effect on function has not been observed. Although studies in tobacco, rice, and tomato reported telomere phenotypes associated with knock-outs or overexpressing Group I TRFL proteins (Yang et al., 2004; Moriguchi et al., 2006; Hong et al., 2007), these effects are relatively mild and may reflect only an auxiliary function of these proteins at telomeres. Instead, these proteins may act as transcription factors as promoters of a number of genes are known to contain a short stretch of telomeric sequences (Tremousaygue et al., 1999). Hence, other proteins likely form the core structure of telomeric chromatin in plants.

The question remains as to what proteins comprise the telomere capping complex in *Arabidopsis*. The Smh/TRB proteins may be the next prime suspects. Phylogenetic analysis shows that these proteins are present in all plant taxonomic units including unicellular green algae suggesting that they may be associated with a fundamental biological function. Three Smh/TRB genes with an N-terminal telobox domain have been found in *Arabidopsis* and have shown to exhibit *in vitro* binding to telomeric DNA (Schrumpfova et al., 2004; Mozgova et al., 2008; Hofr et al., 2009). Recently, *Arabidopsis* TRB1 was found to bind to telomeric sequences *in vivo* through immunolocalization studies in tobacco cells (Schrumpfova et al., 2014). One caveat with this approach is that telomeres in tobacco reach far greater lengths than with *Arabidopsis* (∼5 and 150 kb respectively). Association with telomeric DNA may, therefore, not be necessarily for telomere specific functions and can similarly colocalize with non-telomeric sequences. Chromatin Immunoprecipitation (ChIP) studies performed within the same paper, however, confirm binding to telomeric sequences in *Arabidopsis*. With this evident telomere binding capacity and interaction with Pot1b and the N-Terminus of TERT, SMH proteins also show promise as telomere binding components of *Arabidopsis* telomeres (Kuchar and Fajkus, 2004; Schrumpfova et al., 2014). Telomere length defects are also described for *trb1* mutants although the effect is relatively small after five generations of selfing (Schrumpfova et al., 2014). This could mean redundancy amongst the SMH family of proteins. Additionally, it is possible that members of the tested group 1 TRFL proteins are redundant with SMH/TRB proteins. Functional analysis of other members of this family should clarify the role of these proteins in telomere maintenance.

## Author Contributions

NF designed and performed the experiments and wrote the paper. KR designed the experiments, performed phylogenetic analysis and wrote the paper.

## Conflict of Interest Statement

The authors declare that the research was conducted in the absence of any commercial or financial relationships that could be construed as a potential conflict of interest.
