# Supplementary material for: Using Centromere Mediated Genome Elimination to Elucidate the Functional Redundancy of Candidate Telomere Binding Proteins in Arabidopsis thaliana
Source: Front Genet. 2016 Jan 5;6:349. doi: 10.3389/fgene.2015.00349 (PMC4700174; doi:10.3389/fgene.2015.00349)
Supplement: Supplementary file 2 [file Image_1.PDF]

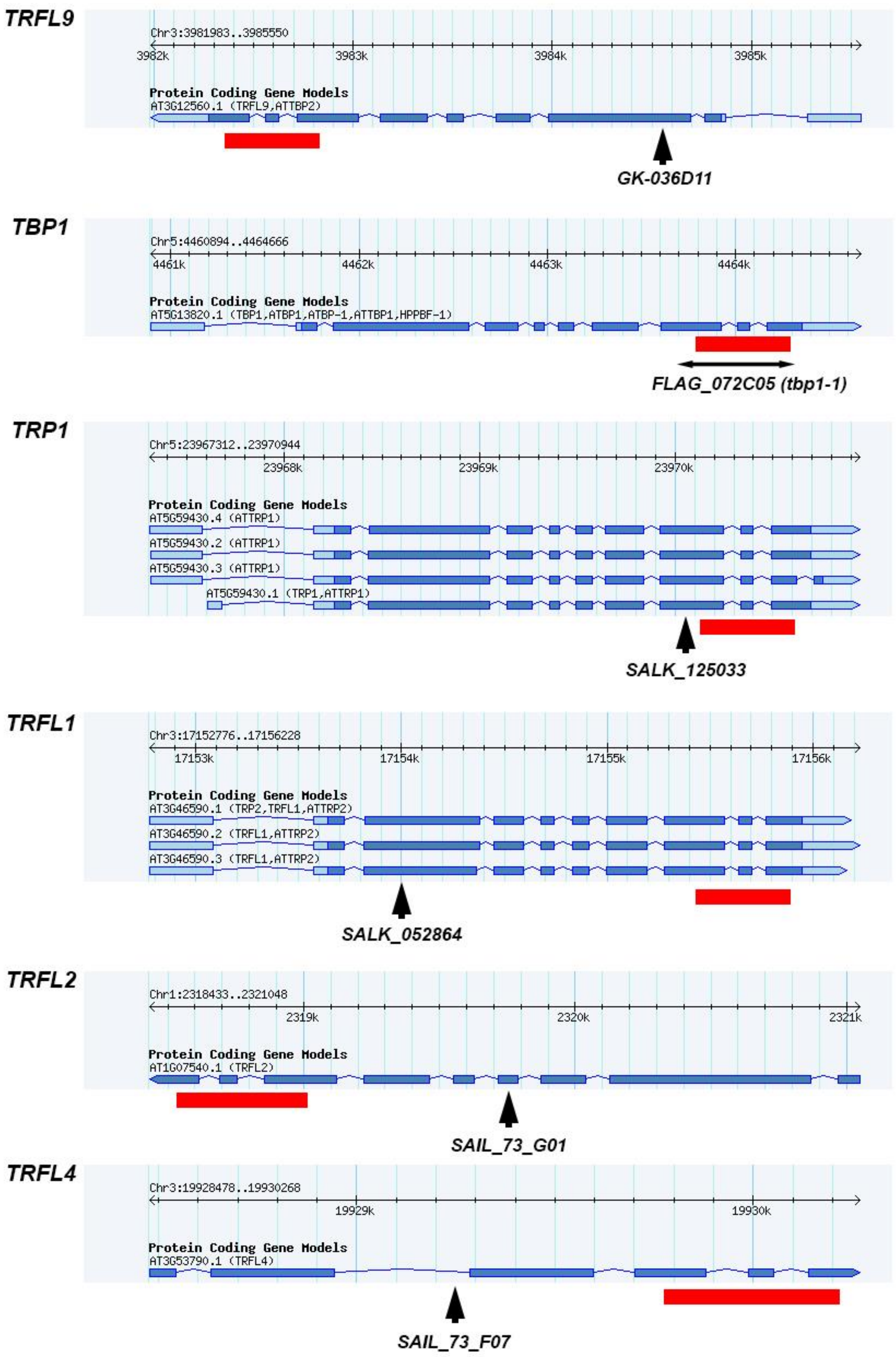

Figure S1. Position of T-DNA insertions in TRFL genes in mutant strains used in this study. Arrows and red boxes indicate position of T-DNA insertions and gene regions encoding Myb-telobox domain, respectively. Orientation of gene models reflects their orientation on a chromosome; the ruler above each gene model shows gene location on a chromosome. Directionality of transcription is indicated by arrowheads at the ends of last exons. The T-DNA insertion in *tbp1-1* allele results in deletion of the entire Myb-telobox domain (Hwang et al., 2007).
